# Splenic artery embolization: technically feasible but not necessarily advantageous

**DOI:** 10.1186/s13017-016-0100-7

**Published:** 2016-09-13

**Authors:** F. Van der Cruyssen, A. Manzelli

**Affiliations:** 1 Third year master’s student, Faculty of Medicine, Catholic University of Leuven (KU Leuven), Gasthuisberg, Belgium; 2 Department of Upper Gastrointestinal Surgery, Royal Devon & Exeter Hospital, Exeter, UK

**Keywords:** Splenic artery, Embolization, Blunt splenic injury, Nonoperative management, Operative splenic salvage, Splenectomy, Trauma

## Abstract

**Background:**

The spleen is the second most commonly injured organ in cases of abdominal trauma. Management of splenic injury depends on the clinical status of the patient and can include nonoperative management (NOM), splenic artery embolization (SAE), surgery (operative splenic salvage or splenectomy), or a combination of these treatments. In nonoperatively managed cases, SAE is sometimes used to control haemorrhage. However, the indications for SAE have not been clearly defined and, in some cases, the potential complications of the procedure may outweigh its benefits.

**Review of the literature:**

Through review of the literature we address the question of when SAE is indicated in combination with NOM of splenic injury, and whether SAE may delay needed surgical treatment in some cases. This systematic review highlighted the use of imperfect and inconsistent scoring systems in the diagnosis of splenic injury, the lack of consensus regarding indications for SAE, and the potential for severe morbidities associated with this procedure. Based on current literature and evidence we provide a new, non-verified, decision algorithm.

**Conclusions:**

NOM+ SAE involves potential risks and operative management may be preferable to SAE for certain patients. To clarify current literature, we propose a new algorithm for blunt abdominal trauma that should be validated prospectively. New evidence-based protocols should be developed to guide diagnosis and management of patients with splenic trauma.

**Electronic supplementary material:**

The online version of this article (doi:10.1186/s13017-016-0100-7) contains supplementary material, which is available to authorized users.

## Background

Abdominal injury occurs in approximately 20–30 % of all trauma patients. The spleen is the second most commonly injured organ in both blunt and penetrating abdominal trauma [1, 2].

Raza et al. reviewed 1285 cases of abdominal trauma over a ten-year period; 26 % of these suffered a splenic injury. Of the patients in the initial study group, 89.91 % were managed without surgery and 30 % of these had a splenic injury. The initial presentation of splenic injury can be variable and depends on the severity of the associated injuries, the amount of blood lost, and the mechanism of trauma. Routine diagnostic workup of suspected splenic injury includes chest radiographs, abdominal ultrasound, a focused assessment with sonography for trauma (FAST) scan, or CT imaging. Abdominal CT imaging with intravenous contrast is the preferred imaging method in hemodynamically stable patients; when the patient is in an unstable condition, a FAST scan that shows the presence of free within the abdomen can indicate that emergency surgery is necessary. Physicians and surgeons should remain alert for concomitant injuries to the liver (the organ most frequently injured by blunt abdominal trauma) and other intra-abdominal organs.

Splenic artery embolization (SAE) is increasingly being used in medical practice. This trend started in the 1970s when Maddison reported the first successful SAE in a patient with esophageal varices, hepatic cirrhosis, and recurrent gastrointestinal bleeding [3]. In 1975, Chuang and Reuter reported successful SAE after splenic injury in ten dogs [4]. In 1985, Sclafani et al. reported that embolization of 107 arterial injuries yielded an 82.2 % success rate [5]. There was a further evolution towards proximal SAE or more distal and selective embolization of the branches of the splenic artery.

Until the 1990s splenectomy remained the treatment of choice for splenic injury, but nonoperative management (NOM) became increasingly popular in the pediatric setting around 1992 when Haller et al. reported on the safety of NOM of solid organ injuries in children [6]. Sclafani reported in 1995 a study of 172 patients and the use angiography with subsequent use of proximal embolization of the splenic artery when extravasation was present [7]. In his study NOM was used in stable patients with no extravasation on angiography, splenectomy or splenorrhaphy was performed in unstable patients or in patients with associated injuries or disease. Overall splenic salvage rate was 88 % with a splenic salvage rate of 97 % in the patients managed non-operatively.

The first large multi-institutional study was published in 2000 by Peitzman et al. [8]. In this EAST study 1488 patients with blunt splenic injury were reviewed. The authors report operative management in 38,5 % of the patients and immediate operative management was proportional to the grade of injury. In patient groups with a larger hemoperitoneum, more laparotomies were performed. They further report a NOM failure rate of 10,8 % of which 60,9 % within the first 24 h of admittance. 54,8 % of the patients were successfully managed nonoperatively.

In 2005 Haan reported on 648 patients in a single institution study with splenic salvage rates of 90 % after SAE and 100 % after planned NOM [9]. Failure of NOM increased with higher injury grades but 80 % of grade IV and V injuries were successfully managed nonoperatively. Interestingly, a 40 % failure rate was reported when an arteriovenous fistula was present, even after embolization.

Currently, between 60 and 80 % of splenic injuries are managed without surgery and the reported success rates for NOM range between 85 and 94 %. NOM can be combined with SAE when certain conditions are met, and retrospective studies have reported higher success rates and higher splenic salvage rates when NOM is combined with SAE [10–12]. However, no consensus has been reached on the correct indications for combining NOM and SAE, especially in higher grade splenic injuries. In the current literature, the use of NOM in combination with SAE for treatment of blunt splenic injury is dependent on multiple factors, which are: the grade of the splenic injury, the injury severity score (ISS), patient age, the presence of contrast blush on CT, active extravasation on angiography, need for transfusion, the presence of significant hemoperitoneum, and the presence of splenic artery pseudoaneurysms. Most trauma protocols consider hemodynamic instability or a high-grade splenic lesion (IV–V) to be indications for emergency laparotomy. Operative splenic salvage (OSS) or splenectomy can then be attempted. However, based on the currently available literature the use of NOM, SAE, or OSS is controversial. Retrospective review of data, small study groups, and changes in protocols or in standard practice make it difficult to draw conclusions regarding the optimal management of splenic injuries.

## Methods & results

We reviewed the literature and used critical interpretive synthesis methodology in the following discussion. A search of the following databases was conducted during October 2015: Medline database, Trip database, EMBASE, Web of Science, Cochrane Library, and Scopus. We also manually searched the reference lists of the retrieved articles. Our search terms included: splenic injury, embolization, operative, management.

We included original research articles that reported on splenic injury in combination with embolization therapy, operative, and nonoperative management. Papers were excluded if they focused specifically on adolescent, pediatric, or neonatal populations and if they included specific diagnostic groups. Guidelines, surveys, and meta-analyses were also excluded. The search was executed sequentially and is documented in Table 1. All articles were assessed for relevance by reading the abstract and, where needed, the entire paper using the above criteria.

**Table 1:** Search results and number of articles retrieved after applying the selection criteria. After the initial search, all articles were entered in a reference database (Mendeley)

Database searched	Search terms used and limits applied	Number of results	Number met inclusion criteria
Medline	((“splenic injury”) AND “embolization”) AND “operative”	50	29
Medline	(((splenic trauma) AND operative management) AND artery embolization) AND non operative management	15	5
Trip database	(title:splenic injury)(title:embolization)(operative)	8	7
EMBASE: Search 1	‘splenic injury’/exp OR ‘splenic injury’ AND (‘embolization’/exp OR embolization) AND operative	90	46
EMBASE: Search 2	#1 AND ([adolescent]/lim OR [adult]/lim OR [young adult]/lim)	46	26
EMBASE: Search 3	#2 AND (‘clinical article’/de OR ‘clinical trial’/de OR ‘comparative study’/de OR ‘controlled study‘/de OR ‘major clinical study‘/de OR ‘medical record review‘/de OR ‘multicenter study‘/de OR ‘observational study’/de OR ‘outcomes research’/de OR ‘prospective study’/de OR ‘retrospective study’/de) AND (‘article’/it OR ‘review’/it)	26	21
Web of Science	TOPIC: (splenic injury) AND TOPIC: (embolization) AND TOPIC: (operative) Timespan: All years. Indexes: SCI-EXPANDED, SSCI, A&HCI, CPCI-S, CPCI-SSH.	91	45
Cochrane Library	splenic injury embolization	7	6
Cochrane Library	splenic injury operative	1	0
Scopus	TITLE-ABS-KEY (splenic injury) AND TITLE-ABS-KEY (embolization) AND TITLE-ABS-KEY (operative)	79	34
Manual search from reference list of retrieved articles	Number in reference list - 1 - 7 - 8 - 9 - 14 - 15 - 16 - 18 - 24 - 30 - 38 - 41 - 42 - 51 Not selected - Hartnett KL, Winchell RJ, Clark DE (2003) Management of adult splenic injury: a 20-year perspective. Am Surg 69:608–611. - Upadhyaya P (2003) Conservative management of splenic trauma: history and current trends. Pediatr Surg Int 19:617–627. - Todd SR, Arthur M, Newgard C et al. (2004) Hospital factors associated with splenectomy for splenic injury: a national perspective. J Trauma 57:1065–1071. - Galvan DA, Peitzman AB (2006) Failure of nonoperative management of abdominal solid organ injuries. Curr Opin Crit Care 12:590–594. - Watson GA, Rosengart MR, Zenati MS et al. (2006) Nonoperative management of severe BSI: are we getting better? J Trauma 61:1113–1119. - Sclafani SJ, Shaftan GW, Scalea TM et al. (1995) Nonoperative salvage of computed tomography-diagnosed splenic injuries: utilization of angiography for triage and embolization for hemostasis. J Trauma 39:818–827.	22	14
TOTAL		435	233

Duplicates were excluded and the final selection of articles was based on their relevance and quality. Papers were excluded if no full text could be obtained. To select high-quality studies, we assessed all articles following the Critical Review Form for Quantitative Studies and guidelines from Law et al. [13]. An overview of the selected articles is presented in Table 2. Excluded articles are also presented in Table 2 for reference. Despite the large amount of articles retrieved, we were not able to carry out a meta-analysis because of the heterogeneity of study methods, data and quality of articles.

**Table 2:** Summary table of articles that met inclusion criteria after initial selection. Articles marked in bold were excluded

	Reference	Study design	Sample size and sites	Comments/key findings	Included/excluded
1	A.P. E, B. I, M. R, M.C. M. The impact of splenic artery embolization on the management of splenic trauma: an 8-year review. *Am J Surg*. 2009	Retrospective study	304 + 416 Single center	4 years NOM versus 4 years NOM + SAE	Included
**2**	**Akinkuolie AA, Lawal OO, Arowolo OA, Agbakwuru EA, Adesunkanmi ARK. Determinants of splenectomy in splenic injuries following blunt abdominal trauma.** ***SOUTH AFRICAN J Surg*** **. 2010**	**Retrospective study**	**55** **Single center**	**Poor overall quality, 1998–2007, small study group**	**Excluded**
**3**	**Albrecht RM, Schermer CR, Morris A. Nonoperative management of blunt splenic injuries: factors influencing success in age >55 years. ** ***Am Surg*** **. 2002**	**Retrospective study**	**37** **Single center**	**Small study group**	**Excluded**
**4**	**Bala M, Edden Y, Mintz Y, et al. Blunt splenic trauma: Predictors for successful non-operative management. ** ***Isr Med Assoc J*** **. 2007**	**Prospective study**	**64** **Single center**	**Admission systolic bloodpressure, extra-abdominal injury are predictors for succesfull NOM, small study group**	**Excluded**
5	Barquist ES, Pizano LR, Feuer W, et al. Inter- and intrarater reliability in computed axial tomographic grading of splenic injury: Why so many grading scales? *J TRAUMA-INJURY Infect Crit CARE*. 2004	Retrospective study	200 Single center	200 CT images were reviewed for inter- and intrarater reliability	Included
**6**	**Benissa N, Boufettal R, Kadiri Y, et al. Non operative management of blunt splenic trauma in adults. ** ***J Chir (Paris)*** **. 2008**	**Retrospective study**	**62** **Single center**	**Overall poor quality paper**	**Excluded**
**7**	**Bhangu A, Nepogodiev D, Lal N, Bowley DM. Meta-analysis of predictive factors and outcomes for failure of non-operative management of blunt splenic trauma.** ***Inj J CARE Inj*** **. 2012**	**Meta-analysis**		**Meta-analysis**	**Excluded**
**8**	**Brillantino A, Iacobellis F, Robustelli U, et al. Non operative management of blunt splenic trauma: a prospective evaluation of a standardized treatment protocol.** ***Eur J Trauma Emerg Surg*** **. September 2015**	**Prospective**	**87**	**No full text available, epub ahead of print**	**Excluded**
**9**	**Brugère C, Arvieux C, Dubuisson V, et al. Early embolisation in non-operative management of blunt splenic injuries: a retrospective multicentric study. ** ***J Chir (Paris)*** **. 2008**	**Retrospective multicentric study**	**22**	**Full text no longer available, Use of Moore clasification, low power**	**Excluded**
10	C. R, A. A, G.P. S, et al. Management of splenic trauma: a single institution’s 8-year experience. *Am J Surg*. 2015	Retrospective registry review	926		Included
11	Chastang L, Bège T, Prudhomme M, et al. Is non-operative management of severe blunt splenic injury safer than embolization or surgery? Results from a French prospective multicenter study. *J Chir Viscerale*. 2015	Prospective multicentric study	91		Included
**12**	**Clancy AA, Tiruta C, Ashman D, Ball CG, Kirkpatrick AW. The song remains the same although the instruments are changing: complications following selective nonoperative management of blunt spleen trauma: A retrospective review of patients at a level I trauma centre from 1996 to 2007**	**Retrospective**	**538**	**Single center study from 1996–2007, lack of data and statistical analysis**	**Excluded**
13	Claridge JA, Carter JW, McCoy AM, Malangoni MA. In-house direct supervision by an attending is associated with differences in the care of patients with a blunt splenic injury. *Surgery*. 2011	Retrospective review	506		Included
14	Cohn SM, Arango JI, Myers JG, et al. Computed Tomography Grading Systems Poorly Predict the Need for Intervention after Spleen and Liver Injuries. *Am Surg*. 2009		300		Included
15	Cooney R, Ku J, Cherry R, et al. Limitations of splenic angioembolization in treating blunt splenic injury. *J Trauma - Inj Infect Crit Care*. 2005	Retrospective	194		Included
16	D. D, G. A, B.A. E, et al. Blunt splenic injuries: High nonoperative management rate can be achieved with selective embolization. *J Trauma - Inj Infect Crit Care*. 2004	Retrospective study	233 + 168		Included
17	D.C. O, J.S.K. L, P.P. DR, et al. Variation in treatment of blunt splenic injury in Dutch academic trauma centers. *J Surg Res*. 2015	Retrospective study	253		Included
18	Dehli T, Bagenholm A, Trasti NC, et al. The treatment of spleen injuries: a retrospective study. *Scand J TRAUMA Resusc Emerg Med*. 2015	Retrospective study	109	More splenic salvage after introduction of SAE	Included
19	Ekeh AP, Khalaf S, Ilyas S, et al. Complications arising from splenic artery embolization: A review of an 11-year experience. *Am J Surg*. 2013	Retrospective study	1383		Included
**20**	**Ekeh AP, McCarthy MC, Woods RJ, et al. Complications arising from splenic embolization after blunt splenic trauma. ** ***Am J Surg*** **. 2005**	**Retrospective study**	**284**	**More recent studies were used.**	**Excluded**
21	Fu C-Y, Wu S-C, Chen R-J, et al. Evaluation of need for operative intervention in blunt splenic injury: intraperitoneal contrast extravasation has an increased probability of requiring operative intervention. *World J Surg*. 2010	Retrospective study	69		Included
22	G. T, E. B, A. B, et al. Nonoperative management of blunt splenic injury in adults: there is (still) a long way to go. The results of the Bologna-Maggiore Hospital trauma center experience and development of a clinical algorithm. *Surg Today*. 2015	Retrospective study	293	Development of a BSI protocol	Included
23	Gaarder C, Dormagen JB, Eken T, et al. Nonoperative management of splenic injuries: improved results with angioembolization. *J Trauma*. 2006	Prospective study compared to historic control group	61 + 64	Results after protocol implementation	Included
24	Gonzalez M, Bucher P, Ris F, Andereggen E, Morel P. Splenic trauma: predictive factors for failure of non-operative management. *J Chir (Paris)*. 2008	Retrospective study	190	Predictive factors	Included
25	Haan JM, Biffl W, Knudson MM, et al. Splenic Embolization Revisited: A Multicenter Review. *J Trauma - Inj Infect Crit Care*. 2004	Retrospective multicentric study	140	Complications SAE	Included
**26**	**Hsieh T-M, Tsai TC, Liang J-L, Lin CC. Non-operative management attempted for selective high grade blunt hepatosplenic trauma is a feasible strategy. ** ***WORLD J Emerg Surg*** **. 2014**	**Retrospective study**	**150**	**Hepatosplenic group**	**Excluded**
27	J. F, M. R, C. A, et al. Blunt splenic injury: are early adverse events related to trauma, nonoperative management, or surgery? *DIAGNOSTIC Interv Radiol*. 2015	Retrospective study	136	OM worse outcomes but related to ISS	Included
28	J. S, T.L. T, J.B. D, et al. Preserved splenic function after angioembolisation of high grade injury. *Injury*. 2012	Retrospective study	58		Included
**29**	**K.K. T, M.T. C, A. V, Tan KK, Chiu MT, Vijayan A. Management of isolated splenic injuries after blunt trauma: An institution’s experience over 6 years.** ***Med J Malaysia*** **. 2010**		**42**	**Did not meet Critical Review Form requirements**	**Excluded**
30	Koca B, Topgul K, Yuruker SS, Cinar H, Kuru B. Non-operative treatment approach for blunt splenic injury: is grade the unique criterion? *Ulus TRAVMA VE ACIL CERRAHI DERGISI-TURKISH J TRAUMA Emerg Surg*. 2013	Retrospective study	31	Factors to consider NOM	Included
**31**	**Koo T-Y, Ra Y-M, Lee SE, et al. Extension of Nonoperative Management on Spleen Injury with Judicious Selection and Embolization; 10 Years of Experience. ** ***J KOREAN Surg Soc*** **. 2011**	**Retrospective study**	**151**	**Lack of statistical analysis, did not meet Critical Review Form requirements**	**Excluded**
**32**	**Kourabi M, Reibel N, Perez M, Grosdidier G. A serious late complication of non-operative management of splenic trauma: rupture of splenic artery aneurysm.** ***J Chir (Paris)*** **. 2008**	**Three case reports**	**3**	**Case reports**	**Excluded**
33	L.A. O, D. S, C.M. D, et al. Implications of the “contrast blush” finding on computed tomographic scan of the spleen in trauma. *J Trauma - Inj Infect Crit Care*. 2001	Retrospective study	324	Contrast blush alone should not mandate management	Included
**34**	**Le Moine M-C, Aguilar E, Vacher C, et al. Splenic injury: Management in the Languedoc-Roussillon region. Survey of public hospital surgeons.** ***J Chir Viscerale*** **. 2010**	**Survey**	**/**	**Surveys are not considered**	**Excluded**
**35**	**Liu PP, Lee WC, Cheng YF, et al. Use of splenic artery embolization as an adjunct to nonsurgical management of blunt splenic injury. ** ***J Trauma*** **. 2004**	**Retrospective?**	**39**	**Did not meet Critical Review Form requirements**	**Excluded**
**36**	**Lutz N, Mahboubi S, Nance ML, Stafford PW. The significance of contrast blush on computed tomography in children with splenic injuries. ** ***J Pediatr Surg*** **. 2004**	**Retrospective study**	**133**	**Paediatric population, blush does not mandate SAE**	**Excluded**
37	Marmery H, Shanmuganathan K, Alexander MT, Mirvis SE. Optimization of selection for nonoperative management of blunt splenic injury: Comparison of MDCT grading systems. *Am J Roentgenol*. 2007	Retrospective observational study	496	Comparison of grading systems	Included
38	Marmorale C, Guercioni G, Siquini W, et al. Non-operative management of blunt abdominal injuries. *Chir Ital*. 2007	Retrospective study	123	Nonspecific patient group, low statistical power	Excluded
**39**	**Matsushima K, Kulaylat AN, Won EJ, Stokes AL, Schaefer EW, Frankel HL. Variation in the management of adolescent patients with blunt abdominal solid organ injury between adult versus pediatric trauma centers: an analysis of a statewide trauma database. ** ***J Surg Res*** **. 2013**	**Retrospective study**	**1532**	**Paediatric study group**	**Excluded**
**40**	**Mayglothling JA, Haan JM, Scalea TM, J.A. M, J.M. H, T.M. S. Blunt splenic injuries in the adolescent trauma population: The role of angiography and embolization. ** ***J Emerg Med*** **. 2009**	**Retrospective study**	**97**	**Adolescent study group**	**Excluded**
**41**	**Mikocka-Walus A, Beevor HC, Gabbe B, Gruen RL, Winnett J, Cameron P. Management of spleen injuries: the current profile. ** ***ANZ J Surg*** **. 2010**	**Retrospective study**	**318**	**Unrepresentative patient population**	**Excluded**
42	Miller PR, Chang MC, Hoth JJ, et al. Prospective Trial of Angiography and Embolization for All Grade III to V Blunt Splenic Injuries: Nonoperative Management Success Rate Is Significantly Improved. *J Am Coll Surg*. 2014	Prospective study	168	Prospective use of angiography and SAE	Included
43	Olthof DCC, Sierink JCC, van Delden OMM, Luitse JSKSK, Goslings JCC. Time to intervention in patients with splenic injury in a Dutch level 1 trauma centre. *Inj J CARE Inj*. 2014	Retrospective study	96	Time to intervention	Included
44	Olthof DC, Joosse P, Bossuyt PMM, et al. Observation Versus Embolization in Patients with Blunt Splenic Injury After Trauma: A Propensity Score Analysis. *World J Surg*. December 2015	Propensity score analysis	206	Use of propensity score to contemperous patient groups	Included
45	Olthof DC, van der Vlies CHCH, van der Vlies CHCH, et al. Consensus strategies for the nonoperative management of patients with blunt splenic injury: A Delphi study. *J Trauma Acute Care Surg*. 2013	Delphi study between 30 experts	N/A		Included
46	P. R, T. G, B. S, et al. Management of blunt injuries to the spleen. *Br J Surg*. 2010	Retrospective study	206	Succes of NOM, age	Included
47	Parihar ML, Kumar A, Gamanagatti S, et al. Role of splenic artery embolization in management of traumatic splenic injuries: a prospective study. *Indian J Surg*. 2013	Prospective study	67	Prospective study of success rates with NOM	Included
**48**	**Ransom KJ, Kavic MS. Laparoscopic splenectomy for blunt trauma: a safe operation following embolization.** ***Surg Endosc OTHER Interv Tech*** **. 2009**	**Retrospective study**	**46**	**Laparoscopic splenectomy is safe, not considered for this review**	**Excluded**
**49**	**Requarth JA. Distal Splenic Artery Hemodynamic Changes During Transient Proximal Splenic Artery Occlusion in Blunt Splenic Injury Patients: A Mechanism of Delayed Splenic Hemorrhage. ** ***J Trauma Inj Infect Crit Care*** **. 2010**	**Retrospective study**	**7**	**Distal versus proximal embolization, lack of statistical power**	**Excluded**
50	S.-C. W, R.-J. C, A.D. Y, et al. Complications associated with embolization in the treatment of blunt splenic injury. *World J Surg*. 2008	Retrospective study	152	Complications of SAE	Included
51	Sabe AA, Claridge JA, Rosenblum DI, Lie K, Malangoni MA. The effects of splenic artery embolization on nonoperative management of blunt splenic injury: a 16-year experience. *J Trauma*. 2009	Retrospective study	815	Three groups, more success NOM when combined with SAE	Included
52	Shih H-C, Wang C-Y, Wen Y-S, et al. Spleen artery embolization aggravates endotoxin hyporesponse of peripheral blood mononuclear cells in patients with spleen injury. *J Trauma*. 2010	Observational study	16	Effect of SAE on splenic function	Included
**53**	**Shiping L, Jianyong L, Zhi Z, Yun Z. Management of Traumatic Splenic Rupture in Adults: A Single Center’s Experience in Mainland China. ** ***Hepatogastroenterology*** **. 2014**	**Retrospective study**	**125**	**No full text available**	**Excluded**
**54**	**Skattum J, Loekke RJV, Titze TL, et al. Preserved function after angioembolisation of splenic injury in children and adolescents: A case control study. ** ***Inj J CARE Inj*** **. 2014**	**Case control**	**11**	**Pediatric and adolescent study group, case control**	**Excluded**
**55**	**Soo K-M, Lin T-Y, Chen C-W, et al. More Becomes Less: Management Strategy Has Definitely Changed over the Past Decade of Splenic Injury-A Nationwide Population-Based Study. ** ***Biomed Res Int*** **. 2015**	**Retrospective study**	**578**	**Lack of statistical analysis, no added value to article**	**Excluded**
**56**	**Stassen NA, Bhullar I, Cheng JD, et al. Selective nonoperative management of blunt splenic injury: An Eastern Association for the Surgery of Trauma practice management guideline.** ***J Trauma Acute Care Surg*** **. 2012**	**Guideline**	**N/A**	**Guideline**	**Excluded**
57	Wahl WL, Ahrns KS, Chen S, et al. Blunt splenic injury: Operation versus angiographic embolization. *Surgery*. 2004	Retrospective study	164	Factors to consider for indication of SAE versus operative management	Included
58	Wei B, Hemmila MR, Arbabi S, et al. Angioembolization reduces operative intervention for blunt splenic injury. *J Trauma - Inj Infect Crit Care*. 2008	Retrospective study	317	less complications and better outcomes with SAE	Included
59	Wu S-C, Fu C-Y, Muo C-H, Chang Y-J. Splenectomy in trauma patients is associated with an increased risk of postoperative type II diabetes: a nationwide population-based study. *Am J Surg*. 2014	Retrospective study	3723	Increased risk for T2DM	Included
60	Zarzaur BL, Croce MA, Fabian TC. Variation in the Use of Urgent Splenectomy After Blunt Splenic Injury in Adults. *J TRAUMA-INJURY Infect Crit CARE*. 2011	Retrospective study	11.793	Mortality after splenectomy	Included
61	Zarzaur BL, Savage SA, Croce MA, Fabian TC. Trauma center angiography use in high-grade blunt splenic injuries: Timing is everything. *J Trauma Acute Care Surg*. 2014	Retrospective study	10.405	Use of angio and role in splenectomy	Included
62	Zarzaur BL, Kozar R, Myers JG, et al. The splenic injury outcomes trial. *J Trauma Acute Care Surg*. 2015	Prospective observational study	383	Risk of splenectomy after NOM + SAE, importance of blush on CT	Included

**Fig. 1:**
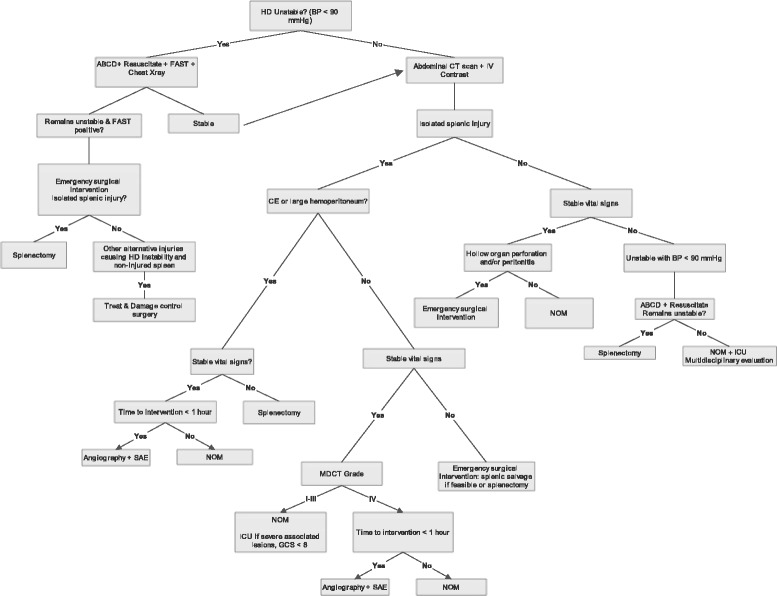
Algorithm for management of splenic trauma modified from Ekeh and Tugnoli [8, 28]. Abbreviations: HD: hemodynamically; BP: blood pressure; FAST: Focused Assessment with Sonography for Trauma; ICU: Intensive Care Unit; SAE: splenic artery embolization; MDCT: Multidetector CT grading (Table 4); NOM: non operative management; CE: contrast extravasation; IV: intravenous

In order to further support the literature review we propose a new, non-verified, algorithm for blunt abdominal trauma, based on existing algorithms presented in the literature and modified according to our findings (Fig. 1).

## Discussion

### Hemodynamically unstable patients

Abdominal trauma with hemodynamic instability is an absolute indication for operative management in most protocols. However, in cases of splenic injury, SAE and NOM is increasingly being performed even in patients that are hemodynamically unstable. The use of NOM or SAE in hemodynamically unstable patients is highly controversial and should be further studied. Delays in diagnostic testing and inappropriate selection of hemodynamically unstable patients for this treatment protocol could increase morbidity and mortality rates, as some authors have suggested [14–16]. Importantly, there has been little research on managing patients with transient hemodynamic stability, although Hagiwara et al. did report successful SAE in 15 patients with transient responses to fluid administration [17].

Most studies in the literature are retrospective case series that describe the immediate technical success of SAE without comparisons with surgical treatment or a purely conservative approach. Operative intervention by means of splenectomy is thought to increase morbidity and mortality rates, but this is biased by higher injury severity scores in patients that are surgically managed. A large study of 11,793 patients by Zarzaur et al. (2011) could not confirm a correlation between splenectomy and in-hospital mortality when the data were corrected for demographic, physiologic, and injury-related factors [18]. The risk of overwhelming post-splenectomy sepsis has been advocated as a concern and relative contraindication for splenectomy; however, this rare complication occurs in only approximately 0.9 % of splenectomy cases and is preventable by immunization [19]. A recent, large, population-based study classified 9719 patients with splenic injuries into two groups, splenectomy or non-splenectomy, and compared these against a control group of 30,413 patients with other abdominal injuries. This study warned of a two-fold increased risk of developing type II diabetes post-splenectomy [20]. In contrast, some authors have reported higher morbidity after SAE than after surgical intervention [21]. In addition, there is evidence of splenic functional alterations after SAE, although research suggests that no immunizations are necessary after NOM combined with SAE [22]. In a 2010 paper, Shih and colleagues demonstrated a cytokine hyporesponse after SAE [23] therefore, SAE should not be considered definitively safer than splenectomy.

### Hemodynamically stable patients

SAE, as opposed to pure conservative management, is even more difficult to advocate in hemodynamically stable patients. In a 2015 study, Olthoff et al. looked at the management of 253 patients with splenic injury in Dutch trauma centers taking into account hemodynamic instability on admission, high-grade injury, and ISS [24]. Although the rate of NOM was comparable between the five trauma centers reviewed, the use of SAE for splenic injuries was highly variable between the centers, illustrating the lack of consensus that exists regarding the management of blunt splenic injury.

In hemodynamically stable patients, retrospective studies have shown that NOM has a reduced failure rate when combined with SAE [12, 25–30], but prospective studies and certain retrospective studies have failed to confirm these findings [11, 15]. Several studies have been published that show increased numbers of both minor and major complications, leading to an increase in time spent in the hospital, with the use of SAE [15, 31–33]. Moreover, there is no consensus on the follow-up management and imaging of patients with splenic injuries. Given the lack of evidence in the literature, Olthof et al. published a study in 2013 using the Delphi method to reach an expert opinion on the optimal management and follow-up protocol for blunt splenic injury [28]. In several questionnaires, trauma surgeons and interventional radiologists have tried to reach consensus on the indications for, and optimal follow-up management and imaging to use with, NOM combined with SAE. Almost all of the experts used the American Association for the Surgery of Trauma (AAST) scoring system to grade splenic injuries (Table 3). They all agreed on the use of NOM with or without SAE in small graded injuries (I–II) with no or only a small hemoperitoneum. In high-grade injuries (III–V) with a large hemoperitoneum and active contrast extravasation half of the experts would still attempt NOM + SAE, but most of them would not make a second attempt if the initial SAE failed. Most importantly, they all agreed that rapid intervention is needed for SAE to succeed. Current recommendations are that intervention should be performed within 60 min of admission in stable patients with active contrast extravasation and within 15 to 30 min when a large hemoperitoneum is present. No consensus was reached on management after failure of the initial NOM, or on the appropriate length of stay (LOS) in the hospital.

**Table 3:** Traditionally used American Association for the Surgery of Trauma (AAST) scoring system for splenic injuries

Grade	Hematoma	Laceration
I	Subcapsular < 10 % of surface area	Capsular tear < 1 cm deep into parenchyma
II	Subcapsular 10–50 % of surface area	Capsular tear 1–3 cm deep into parenchyma NO involvement of trabecular vessels
III	Subcapsular > 50 % of surface area OR Expanding, ruptured subcapsular or parenchymal hematoma OR Intraparenchymal hematoma > 5 cm or expanding	>3 cm deep into parenchyma or trabecular vessel involvement
IV		Segmental or hilar vessel involvement with major devascularization (>25 %) of spleen
V	Shattered spleen	Hilar vascular injury that devascularizes spleen

In search of better criteria for determining the need for intervention, Koca et al. correlated the results of NOM with the American Association for the Surgery of Trauma (AAST) splenic injury grade (see below) [34]. They found that hemodynamically stable patients can safely be treated with NOM, including patients with multi-organ injuries and higher grade splenic injuries. Most injuries are lower grade injuries (I–III), and not surprisingly transfusion is correlated with the injury grade. A 2008 article by Gonzalez et al. added the degree of hemoperitoneum and associated injuries as predictive factors for NOM failure [35]. In 2004, Wahl and colleagues retrospectively analyzed 164 patients with blunt splenic injuries; [36] after univariate analysis they found that lower blood pressure, higher ISS, lower pH, and more packed RBC transfusions are the best indicators of the need for operative intervention. Age, heart rate, splenic abbreviated injury scale score, and GCS did not significantly correlate with the need for operative management. Similarly, Tugnoli et al. (2014) presented the algorithm used in the Bologna-Maggiore Hospital; in 293 patients with splenic injuries admitted between 2009 and 2013 [37] they reported a NOM success rate of 95.8 %. All hemodynamically stable cases with active contrast extravasation or grade V injuries were embolized, preferably proximally within the splenic artery.

In a 2015 AAST prospective observational study, Zarzaur et al. investigated the risk of delayed splenectomy after NOM with or without SAE [38]. They found that the only risk factor for delayed splenectomy was the finding of active extravasation at presentation; thus calling for closer observation of patients with this finding irrespective of whether SAE was performed. Patients with grade I injury had no risk for delayed splenectomy. In cases with higher grade (II to V) injuries, the authors advocated close observation for 10–14 days. In the 2005 study of Haan et al. the presence of arteriovenous fistula on CT imaging was predictive for 40 % of failure rates in nonoperatively managed patients [9]. The presence of vascular lesions (arteriovenous fistulas, pseudoaneurysms) and impact on NOM success rates was further investigated by several authors. In a 2014 study, the Wake Forest University investigated failure rates after angiography with subsequent embolization, ignoring presence or absence of any vascular lesion, for all grade III to V splenic injuries in stable patients in a protocolized manner and comparing this to a historic control group [39]. From 168 patients 67 % were managed nonoperatively with an overall NOM failure rate of 5 %. In the patient group where protocol was violated the failure rate was 25 % compared to 3 % in the protocol group (*P = 0.03*). Comparing this to a historic control group, 52 % patients underwent NOM with a significant higher failure rate of 15 % (*P = 0.04*). For each injury grade they reported lower failure rates in the study group compared to the control group and when protocol was followed. However, they did not reach statistical significance when comparing patients treated according to protocol or patients deviating from protocol in injury grades IV and V. The authors did not report on complications or on splenic function after embolization in their study group, no data is available whether proximal or distal embolization was used.

Another prospective study from Parihar et al. [40] found a shorter LOS in SAE patients (5.4 days compared with 6.6 days for NOM patients without SAE), as well as higher hemoglobin concentrations and systolic blood pressures. A recent study, which used propensity score analysis, found no statistically significant difference between observation and embolization in the subsequent splenectomy rate [41].

### Scoring systems

Many scoring systems have been developed to grade splenic injuries, among which the AAST grading system for splenic injury (Table 3) is the most popular. Two separate studies have correlated CT findings with the AAST injury grade and subsequent need for intervention. In the study by Barquist et al. [42], four radiologists retrospectively reviewed 200 CT images of patients who had undergone splenectomy; the operative grading of the splenic injuries was used as a gold standard. The weighted kappa score for intrarater reproducibility was 0.15–0.77 and the interrater kappa score was 0–0.84 (mean 0.23). In the study by Cohn et al. [43], five radiologists reviewed CT scans of 300 patients with liver and splenic injuries using the AAST grading system. Twenty-one percent of the patients with splenic injuries visible on CT images required intervention. Cohn et al. reached the same conclusion as Barquist et al.; the sensitivity of the AAST injury grade for predicting the need for intervention is poor and interrater variability, even among experienced radiologists, is high. Both thus concluded that the AAST scoring system is unreliable because even experienced radiologists often underestimate the magnitude of the injury, and other factors should also be considered when evaluating indications for surgery or angiography. The authors also evaluated kappa scores for the reporting of contrast blush on CT imaging and found that they were similarly variable, although contrast blush on CT imaging is considered a major indication that intervention is needed.

In 2001, Protetch et al. reported that the incidence of contrast blush was related to the grade of injury, with 3.2 % of grade I/II injuries and up to 37.6 % of grade IV and V injuries showing a contrast blush in CT images [44]. Interventions were more frequent when a contrast blush was present, but multivariate regression analysis showed no correlation between finding a contrast blush and splenic intervention. This was confirmed by both Thompson et al. and Michailidou et al.; both also reported that the size of the contrast blush was correlated with the need for intervention [45, 46]. They both found that similar cut-off values (1 cm and 1.5 cm) indicated the need for intervention. Other factors that also correlated with the need for intervention in splenic injury were injury grade, hypotension on admission to the emergency department, and age. The findings of these studies support the need for better protocols and parameters to allow proper assessment of the need for intervention in cases of splenic trauma.

In 2007, Marmery et al. suggested a new grading system for splenic injury that takes into account active bleeding or splenic vascular injury (including splenic pseudoaneurysms and arteriovenous fistulas) [47]. Their new grading system was statistically significantly better than the AAST grading system in terms of its ability to predict the need for splenic arteriography (*P = 0.0036*) or the combination of arteriography and surgery (*P = 0.0006*). Table 4 presents the proposed new grading system.

**Table 4:** Newly proposed Multi-Detector CT (MDCT) grading system, reproduced from Marmery et al. (2007)

Grade	
I	● Subcapsular hematoma < 1-cm thick ● Laceration < 1 cm deep into parenchyma
II	● Subcapsular hematoma 1–3-cm thick ● Parenchymal hematoma 1–3-cm diameter ● Laceration 1–3 cm deep into parenchyma
III	● Splenic capsular disruption ● Subcapsular hematoma > 3-cm thick ● Parenchymal hematoma > 3-cm diameter ● Laceration > 3 cm deep into parenchyma
IVa	● Active intraparenchymal and subcapsular splenic bleeding ● Splenic vascular injury (pseudoaneurysm or arteriovenous fistula) ● Shattered spleen
IVb	● Active intraperitoneal bleeding

### Complications

There has been much research published, and work is still ongoing, to understand the complications after SAE compared with those after surgery and NOM. All of the treatment modalities for splenic injury are associated with high morbidity rates. Specific morbidities associated with SAE include pancreatitis, splenic infarction, post-embolization syndrome, and intestinal perforation.

Two retrospective studies examined the complications that occur after SAE [32, 33]. Ekeh et al. reviewed 1383 patients with blunt splenic injury over an 11-year period; 78.5 % were treated nonoperatively, and of this group 8.1 % underwent SAE. Major complications including splenic infarction, splenic abscess, contrast-induced renal insufficiency, and splenic cysts occurred in 14 % of the patients who underwent SAE. Wu et al. reported that 28.5 % of patients who underwent SAE experienced major complications. Distal embolization was associated with more major complications than proximal SAE. Minor complications (pleural effusion, coil migration, and fever) occurred in 34 % and 61.9 % of the patients in the SAE groups in the studies by Ekeh et al. and Wu et al., respectively.

In 2004, Haan et al. reported SAE complication rates in 140 patients and reported a splenic salvage rate of 87 % [31]. This rate indirectly correlated with injury severity scores. More than 80 % of the grade IV and V injuries in their study were successfully managed nonoperatively. No significant difference was noted in patients older than 55 years of age. The presence of a significant hemoperitoneum did not alter success rates; however, the presence of arteriovenous fistulas did. They concluded that complications after SAE are common, but do not seem to influence outcomes.

In 2008, Wei et al. published a retrospective study showing lower complication rates after SAE even with higher splenic Abbreviated Injury Scores in this group compared with the operatively managed group (6 % vs. 36 %, *P < 0.01*) [30]. They found that the introduction of SAE reduced operative interventions by 16 % between 2000 and 2006 in their Level 1 center. A French prospective multicenter study investigated complication rates in 91 patients [21]. Twenty percent underwent splenectomy with a post-surgical morbidity of 15 %. Fifteen patients (16 %) underwent embolization and 67 were initially managed nonoperatively. In the embolization group with severe injury (grade III–V), the morbidity was 73 %. The splenectomy group with severe injury had a total morbidity of 70 %. The NOM group had a morbidity of 58 %. Specific morbidities after NOM, surgery, and SAE were 10, 15, and 47 % (*P = 0.02)*, respectively. The study concluded that embolization should not be used as a prophylactic measure, but should only be used in cases with active bleeding. We could not find well-designed studies comparing complication rates between the three treatment modalities.

### Other factors to be considered

- Need for transfusion

In their 2015 study of 253 patients, Olthoff et al. found a very large variation but no significant difference in the need for transfusion between patients treated with SAE and those treated with NOM [24]. The mean number of transfused blood products was 5.5 units (±9.9) in the NOM group versus 9.1 units (±17.2) in embolized patients (*P = 0.75*). Rosati et al. reported on the need for transfusion in patients requiring immediate splenectomy (70 %), embolization (46.5 %), and NOM (25.9 %), but failed to adjust these data for injury severity and confounders [12]. They found that the need for transfusion was a major risk factor for mortality (adjusted OR = 2.63; CI 1.27 – 5.42, *P = 0.009*). Dent et al. compared transfusion rates between NOM, early embolization, and operative management [25]. They reported a 2.1-unit difference in the transfusion rate (*P < 0.01)* of the NOM group versus the operative management group, and found no statistically significant difference between transfusion rates in the early embolization and operative management groups without correcting for confounders. They also found no difference in transfusion rates when they compared patients who underwent early embolization versus late embolization.

- Impact of direct supervision

A retrospective review in 2011 investigated the impact of direct supervision (DS) or indirect supervision (IS) on the management of splenic injuries [48]. Using data from 506 cases the authors found significant differences in compliance with protocols (DS, 95 % vs. IS, 82 %, *P < 0.001*), operation rates (DS 16 %, vs. IS, 8 %, *P = 0.016*), ICU use (IS, 84.1 % vs. DS, 73.0 %, *P = 0.029*), hospital costs (IS, $142.956 ± $36.219 vs. DS, $62.981 ± $8.784, *P = 0.048*), and use of SAE without indication (IS, 8 vs. DS, 0). Interestingly, there were no reported differences in mortality or splenectomy rates.

- Time to intervention and volume of the centers.

In 2014, Olthof et al. retrospectively examined the time to intervention in patients undergoing SAE and patients undergoing surgery in a cohort of 96 adults [49]. In hemodynamically stable patients, the median time to intervention for patients in the SAE group was 117 min compared with 105 min for patients in the surgery group. The differences in time to intervention or re-intervention, and the complication rates were not significantly different between the two patient groups. There was a higher transfusion rate for patients in the splenic surgery group; the median number of transfused units of packed RBCs was eight for hemodynamically unstable patients undergoing SAE versus 24 for patients undergoing splenic surgery (*P = 0.09*). A report published in 2015 reviewed 10,405 records in the National Trauma Data Bank to find a correlation between high and low angio centers and to investigate any relationship between angiography use and its timing or splenectomy after angiography [50]. They concluded that early angiography is associated with higher splenic salvage rates but found no statistically significant difference in splenectomy rates between the high and low angiography volume centers.

We could not find any articles comparing the relationship between time to intervention and success or complication rates between patients undergoing NOM, SAE, or splenic surgery.

- Costs & Length of stay

Different authors have investigated the costs of SAE versus surgical management. Wahl et al. reported no significant cost differences between these two treatment modalities (SAE, $49.300 ± $40.460 vs. OM, $54.590 ± $34.760) [36]. This was confirmed by Wei et al., who found the costs associated with SAE to be $47,000 ± $31,000 and the costs associated with OM to be $40,000 ± $34,000 [30]. Both authors assessed the LOS and differences in LOS in patients who received different treatments. Wahl et al. found an average LOS of 45 ± 26 days for patients who went directly to the operating theatre, compared with 14 ± 15 days for patients who underwent SAE (*P < 0.02*). Wei et al. found that the average LOS in the surgical group was 14 ± 10 days and in the SAE group was 12 ± 12 days (*P > 0.05)*. Bruce et al. also came to the same conclusion in their 2011 study; [51] in that study the median total hospital charges were $41,269 (± $31,128) for the NOM + SAE group compared with $46,356 (± $11,334) for the OM group (*P = 0.*545). They did report higher procedure-related charges for OM patients compared with patients undergoing NOM + SAE ($28,709 ± $6941 vs. $19,062 ± $14,025; *P = 0.16*); however, this was offset by more charges for a greater number of radiological evaluations in the other group.

## Summary

Based on the literature review we propose a new, non-verified, decision algorithm for splenic trauma. We hope this can further serve as a tree of order in the vast amount of publications concerning this topic. We stress that this algorithm is not verified and should be further verified in well designed, prospective studies, is it to be used in daily practice.

The term hemodynamically unstable is not clearly defined in most literature and it is often unclear if authors included or excluded transient responders. The nature of the patient’s response to fluid therapy is important and should be considered in all splenic trauma protocols. Most authors agree that surgical intervention is indicated when the patient is hemodynamically unstable. In close relation with response to fluid resuscitation is time to intervention, which is an important factor to consider from the start. Higher splenic salvage rates are achieved when earlier intervention is possible and expert opinion states that intervention is recommended within one hour of admittance. In prospect, when the time to intervention will be several hours OM or NOM may be better options than SAE, depending on the clinical presentation. Clinicians and surgeons should always keep in mind that pre- and post-intervention delays make it increasingly difficult to operate because of the several surgical and nonsurgical issues that can develop (e.g., clotting of the intraperitoneal hemorrhage, infection, and development of comorbidities). Non responders should be triaged promptly with a minimal delay towards surgical intervention.

There is a distinction between major and minor complications and treatment-specific morbidities. While it is difficult to correlate complications with the embolization procedure, especially in small studies, it is interesting to look at treatment-specific complications and associated risk-benefit ratios; however, no well-designed studies compare NOM, NOM + SAE, and OM for the treatment of splenic trauma. Until such studies are available, we should look at the clinical value and possible risks of an intervention on a patient-specific level while considering all factors, and keeping in mind the relatively high complication rate after SAE. It is important to avoid the bias of expecting operative interventions to have higher complication rates than SAE, which seems like a less invasive procedure. Increased use of NOM with or without SAE should not erode clinical judgment and surgical skills.

Currently there is no evidence of reduced transfusion rates when SAE is used in conjunction with NOM compared with NOM alone. Transfusion rates are higher in patients who undergo an operative intervention and are a risk factor for mortality; however, no studies are available that compare similar patient groups and their corresponding transfusion rates.

Multiple studies have reported on the cost effectiveness of splenic injury management: operative intervention brings the costs of surgery and of higher transfusion rates; however, conversely NOM + SAE requires higher imaging costs and there is ultimately no significant difference between the costs of the two interventions.

We reviewed the different scoring systems and the use of imaging for diagnosing splenic injuries. As imaging modalities improve smaller bleeds are increasingly being visualized. It is known that these bleeds can be self-limiting; however, no studies have shown the clinical significance of these small bleeds and other radiological findings such as pseudoaneurysms and arteriovenous fistulas. Additional studies will be needed to assess whether embolization is superior to NOM in these cases. Studies have shown important interrater and intrarater differences in image evaluation in cases of splenic injury, and more specifically in the assessment of a contrast blush on CT imaging. Contrast blush is considered an important parameter for assessment of the need for intervention but recent studies have shown that the size of the contrast blush is what matters. Though whether a cut-off value of 1 cm or 1.5 cm is more suitable for determining the optimal treatment is not yet known. According to the literature, the presence of a hemoperitoneum does not alter the success rates of SAE.

The MDCT grading system proposed by Marmery et al. has shown a better correlation with the need for intervention than the conventionally used AAST grading system. Currently, there is no consensus on the use of one grading system that correlates well with the need for intervention, and most published studies have used the AAST grading system. Lack of standardized grading makes it difficult to compare older studies with recent and future studies. Interpreting CT images and grading the splenic injury is difficult and should be performed by experienced radiologists using the proposed grading system with a consistent technique. We propose the use of specific image analysis protocols to achieve a consistent approach and improve future studies. We propose the use of the MDCT grading system in our decision algorithm.

The treatment plan should be managed by experienced trauma surgeons or interventional radiologists using a multidisciplinary approach when possible. It should be clear who is responsible for assessing the indications for SAE, NOM, or OM. The literature has shown better results when protocols are in place; and ideally there should be consensus on who is responsible for initiating treatment.

## Conclusions

The current literature is unclear on the correct indications for NOM ± SAE versus surgery for splenic trauma. To further clarify current evidence we propose, a non-verified, decision algorithm for blunt abdominal trauma. A prospective well designed study is needed to validate our decision algorithm. Consensus on the management of blunt splenic injury between radiologists, trauma surgeons and emergency medicine physicians could reduce conflicting data, improve current protocols, and avoid harm to patients. More prospective data and well-designed studies, taking into account other factors including long term results and morbidity, on the management of splenic trauma are needed.

## Additional file

Additional file 1: Table 1. Search results and number of articles retrieved after applying the selection criteria.After the initial search, all articles were entered in a reference database (Mendeley). **Table 2.** Summary table of articles that met inclusion criteria after initial selection. Articles marked in grey were excluded. **Table 3.** Traditionally used American Association for the Surgery of Trauma (AAST) scoring system for splenic injuries. **Figure 1.** Algorithm for management of splenic trauma modified from Ekeh and Tugnoli.8, 32 Abbreviations: HD: hemodynamically; BP: blood pressure; FAST: Focused Assessment with Sonography for Trauma; ICU: Intensive Care Unit; SAE: splenic artery embolization; MDCT: Multidetector CT grading (Table 4); NOM: non operative management; CE: contrast extravasation; IV: intravenous. (ZIP 126 kb)

## Abbreviations

AAST: American Association for the Surgery of Trauma
BP: Blood pressure
CE: Contrast extravasation
DS: Direct supervision
FAST scan: Focused assessment with sonography for trauma
GCS: Glasgow coma scale
HD: Hemodynamically
ICU: Intensive care unit
IS: Indirect supervision
ISS: Injury severity score
IV: Intravenous
LOS: Length of stay
MDCT: Multidetector computer tomography
NOM: Nonoperative management
OM: Operative management
OR: Operating room
OSS: Operative splenic salvage
RBCs: Red blood cells
SAE: Splenic artery embolization
