# Supplementary material for: Splenic artery embolization: technically feasible but not necessarily advantageous
Source: World J Emerg Surg. 2016 Sep 13;11(1):47. doi: 10.1186/s13017-016-0100-7 (PMC5020467; doi:10.1186/s13017-016-0100-7)
Supplement: Additional file 1: Table 1. — Search results and number of articles retrieved after applying the selection criteria.After the initial search, all articles were entered in a reference database (Mendeley). Table 2. Summary table of articles that met inclusion criteria after initial selection. Articles marked in grey were excluded. Table 3. Traditionally used American Association for the Surgery of Trauma (AAST) scoring system for splenic injuries. Figure 1. Algorithm for management of splenic trauma modified from Ekeh and Tugnoli.8, 32 Abbreviations: HD: hemodynamically; BP: blood pressure; FAST: Focused Assessment with Sonography for Trauma; ICU: Intensive Care Unit; SAE: splenic artery embolization; MDCT: Multidetector CT grading (Table 4); NOM: non operative management; CE: contrast extravasation; IV: intravenous. (ZIP 126 kb) [file 13017_2016_100_MOESM1_ESM.zip › Table 3.docx]

**Table 3**. Traditionally used American Association for the Surgery of Trauma (AAST) scoring system for splenic injuries.

| **Grade** | **Hematoma** | **Laceration** |
| --- | --- | --- |
| I | Subcapsular < 10% of surface area | Capsular tear < 1 cm deep into parenchyma |
| II | Subcapsular 10%–50% of surface area | Capsular tear 1–3 cm deep into parenchyma NO involvement of trabecular vessels |
| III | Subcapsular > 50% of surface area  OR  Expanding, ruptured subcapsular or parenchymal hematoma  OR  Intraparenchymal hematoma > 5 cm or expanding | > 3 cm deep into parenchyma  or  trabecular vessel involvement |
| IV |  | Segmental or hilar vessel involvement with major devascularization (> 25%) of spleen |
| V | Shattered spleen | Hilar vascular injury that devascularizes spleen |
