# Supplementary material for: Splenic artery embolization: technically feasible but not necessarily advantageous
Source: World J Emerg Surg. 2016 Sep 13;11(1):47. doi: 10.1186/s13017-016-0100-7 (PMC5020467; doi:10.1186/s13017-016-0100-7)
Supplement: Additional file 1: Table 1. — Search results and number of articles retrieved after applying the selection criteria.After the initial search, all articles were entered in a reference database (Mendeley). Table 2. Summary table of articles that met inclusion criteria after initial selection. Articles marked in grey were excluded. Table 3. Traditionally used American Association for the Surgery of Trauma (AAST) scoring system for splenic injuries. Figure 1. Algorithm for management of splenic trauma modified from Ekeh and Tugnoli.8, 32 Abbreviations: HD: hemodynamically; BP: blood pressure; FAST: Focused Assessment with Sonography for Trauma; ICU: Intensive Care Unit; SAE: splenic artery embolization; MDCT: Multidetector CT grading (Table 4); NOM: non operative management; CE: contrast extravasation; IV: intravenous. (ZIP 126 kb) [file 13017_2016_100_MOESM1_ESM.zip › Table 2.docx]

**Table 2.** Summary table of articles that met inclusion criteria after initial selection. Articles marked in grey were excluded.

|  | **Reference** | **Study Design** | **Sample size and sites** | **Comments/key findings** | **Included/excluded** |
| --- | --- | --- | --- | --- | --- |
| 1 | A.P. E, B. I, M. R, M.C. M. The impact of splenic artery embolization on the management of splenic trauma: an 8-year review. *Am J Surg*. 2009 | Retrospective study | 304 + 416  Single center | 4 years NOM versus 4 years NOM+SAE | Included |
| 2 | Akinkuolie AA, Lawal OO, Arowolo OA, Agbakwuru EA, Adesunkanmi ARK. Determinants of splenectomy in splenic injuries following blunt abdominal trauma. *SOUTH AFRICAN J Surg*. 2010 | Retrospective study | 55  Single center | Poor overall quality, 1998-2007, small study group | Excluded |
| 3 | Albrecht RM, Schermer CR, Morris A. Nonoperative management of blunt splenic injuries: factors influencing success in age >55 years. *Am Surg*. 2002 | Retrospective study | 37  Single center | Small study group | Excluded |
| 4 | Bala M, Edden Y, Mintz Y, et al. Blunt splenic trauma: Predictors for successful non-operative management. *Isr Med Assoc J*. 2007 | Prospective study | 64  Single center | Admission systolic bloodpressure, extra-abdominal injury are predictors for succesfull NOM, small study group | Excluded |
| 5 | Barquist ES, Pizano LR, Feuer W, et al. Inter- and intrarater reliability in computed axial tomographic grading of splenic injury: Why so many grading scales? *J TRAUMA-INJURY Infect Crit CARE*. 2004 | Retrospective study | 200  Single center | 200 CT images were reviewed for inter- and intrarater reliability | Included |
| 6 | Benissa N, Boufettal R, Kadiri Y, et al. Non operative management of blunt splenic trauma in adults. *J Chir (Paris)*. 2008 | Retrospective study | 62  Single center | Overall poor quality paper | Excluded |
| 7 | Bhangu A, Nepogodiev D, Lal N, Bowley DM. Meta-analysis of predictive factors and outcomes for failure of non-operative management of blunt splenic trauma. *Inj J CARE Inj*. 2012 | Meta-analysis |  | Meta-analysis | Excluded |
| 8 | Brillantino A, Iacobellis F, Robustelli U, et al. Non operative management of blunt splenic trauma: a prospective evaluation of a standardized treatment protocol. *Eur J Trauma Emerg Surg*. September 2015 | Prospective | 87 | No full text available, epub ahead of print | Excluded |
| 9 | Brugère C, Arvieux C, Dubuisson V, et al. Early embolisation in non-operative management of blunt splenic injuries: a retrospective multicentric study. *J Chir (Paris)*. 2008 | Retrospective multicentric study | 22 | Full text no longer available, Use of Moore clasification, low power | Excluded |
| 10 | C. R, A. A, G.P. S, et al. Management of splenic trauma: a single institution’s 8-year experience. *Am J Surg*. 2015 | Retrospective registry review | 926 |  | Included |
| 11 | Chastang L, Bège T, Prudhomme M, et al. Is non-operative management of severe blunt splenic injury safer than embolization or surgery? Results from a French prospective multicenter study. *J Chir Viscerale*. 2015 | Prospective multicentric study | 91 |  | Included |
| 12 | Clancy AA, Tiruta C, Ashman D, Ball CG, Kirkpatrick AW. The song remains the same although the instruments are changing: complications following selective nonoperative management of blunt spleen trauma: A retrospective review of patients at a level I trauma centre from 1996 to 2007 | Retrospective | 538 | Single center study from 1996-2007, lack of data and statistical analysis | Excluded |
| 13 | Claridge JA, Carter JW, McCoy AM, Malangoni MA. In-house direct supervision by an attending is associated with differences in the care of patients with a blunt splenic injury. *Surgery*. 2011 | Retrospective review | 506 |  | Included |
| **14** | Cohn SM, Arango JI, Myers JG, et al. Computed Tomography Grading Systems Poorly Predict the Need for Intervention after Spleen and Liver Injuries. *Am Surg*. 2009 |  | 300 |  | Included |
| 15 | Cooney R, Ku J, Cherry R, et al. Limitations of splenic angioembolization in treating blunt splenic injury. *J Trauma - Inj Infect Crit Care*. 2005 | Retrospective | 194 |  | Included |
| 16 | D. D, G. A, B.A. E, et al. Blunt splenic injuries: High nonoperative management rate can be achieved with selective embolization. *J Trauma - Inj Infect Crit Care*. 2004 | Retrospective study | 233+168 |  | Included |
| **17** | D.C. O, J.S.K. L, P.P. DR, et al. Variation in treatment of blunt splenic injury in Dutch academic trauma centers. *J Surg Res*. 2015 | Retrospective study | 253 |  | Included |
| 18 | Dehli T, Bagenholm A, Trasti NC, et al. The treatment of spleen injuries: a retrospective study. *Scand J TRAUMA Resusc Emerg Med*. 2015 | Retrospective study | 109 | More splenic salvage after introduction of SAE | Included |
| 19 | Ekeh AP, Khalaf S, Ilyas S, et al. Complications arising from splenic artery embolization: A review of an 11-year experience. *Am J Surg*. 2013 | Retrospective study | 1383 |  | Included |
| 20 | Ekeh AP, McCarthy MC, Woods RJ, et al. Complications arising from splenic embolization after blunt splenic trauma. *Am J Surg*. 2005 | Retrospective study | 284 | More recent studies were used. | Excluded |
| **21** | Fu C-Y, Wu S-C, Chen R-J, et al. Evaluation of need for operative intervention in blunt splenic injury: intraperitoneal contrast extravasation has an increased probability of requiring operative intervention. *World J Surg*. 2010 | Retrospective study | 69 |  | Included |
| **22** | G. T, E. B, A. B, et al. Nonoperative management of blunt splenic injury in adults: there is (still) a long way to go. The results of the Bologna-Maggiore Hospital trauma center experience and development of a clinical algorithm. *Surg Today*. 2015 | Retrospective study | 293 | Development of a BSI protocol | Included |
| 23 | Gaarder C, Dormagen JB, Eken T, et al. Nonoperative management of splenic injuries: improved results with angioembolization. *J Trauma*. 2006 | Prospective study compared to historic control group | 61+64 | Results after protocol implementation | Included |
| 24 | Gonzalez M, Bucher P, Ris F, Andereggen E, Morel P. Splenic trauma: predictive factors for failure of non-operative management. *J Chir (Paris)*. 2008 | Retrospective study | 190 | Predictive factors | Included |
| 25 | Haan JM, Biffl W, Knudson MM, et al. Splenic Embolization Revisited: A Multicenter Review. *J Trauma - Inj Infect Crit Care*. 2004 | Retrospective multicentric study | 140 | Complications SAE | Included |
| 26 | Hsieh T-M, Tsai TC, Liang J-L, Lin CC. Non-operative management attempted for selective high grade blunt hepatosplenic trauma is a feasible strategy. *WORLD J Emerg Surg*. 2014 | Retrospective study | 150 | Hepatosplenic group | Excluded |
| 27 | J. F, M. R, C. A, et al. Blunt splenic injury: are early adverse events related to trauma, nonoperative management, or surgery? *DIAGNOSTIC Interv Radiol*. 2015 | Retrospective study | 136 | OM worse outcomes but related to ISS | Included |
| 28 | J. S, T.L. T, J.B. D, et al. Preserved splenic function after angioembolisation of high grade injury. *Injury*. 2012 | Retrospective study | 58 |  | Included |
| 29 | K.K. T, M.T. C, A. V, Tan KK, Chiu MT, Vijayan A. Management of isolated splenic injuries after blunt trauma: An institution’s experience over 6 years. *Med J Malaysia*. 2010 |  | 42 | Did not meet Critical Review Form requirements | Excluded |
| **30** | Koca B, Topgul K, Yuruker SS, Cinar H, Kuru B. Non-operative treatment approach for blunt splenic injury: is grade the unique criterion? *Ulus TRAVMA VE ACIL CERRAHI DERGISI-TURKISH J TRAUMA Emerg Surg*. 2013 | Retrospective study | 31 | Factors to consider NOM | Included |
| 31 | Koo T-Y, Ra Y-M, Lee SE, et al. Extension of Nonoperative Management on Spleen Injury with Judicious Selection and Embolization; 10 Years of Experience. *J KOREAN Surg Soc*. 2011 | Retrospective study | 151 | Lack of statistical analysis, did not meet Critical Review Form requirements | Excluded |
| 32 | Kourabi M, Reibel N, Perez M, Grosdidier G. A serious late complication of non-operative management of splenic trauma: rupture of splenic artery aneurysm. *J Chir (Paris)*. 2008 | Three case reports | 3 | Case reports | Excluded |
| **33** | L.A. O, D. S, C.M. D, et al. Implications of the “contrast blush” finding on computed tomographic scan of the spleen in trauma. *J Trauma - Inj Infect Crit Care*. 2001 | Retrospective study | 324 | Contrast blush alone should not mandate management | Included |
| 34 | Le Moine M-C, Aguilar E, Vacher C, et al. Splenic injury: Management in the Languedoc-Roussillon region. Survey of public hospital surgeons . *J Chir Viscerale*. 2010 | Survey | / | Surveys are not considered | Excluded |
| 35 | Liu PP, Lee WC, Cheng YF, et al. Use of splenic artery embolization as an adjunct to nonsurgical management of blunt splenic injury. *J Trauma*. 2004 | Retrospective? | 39 | Did not meet Critical Review Form requirements | Excluded |
| 36 | Lutz N, Mahboubi S, Nance ML, Stafford PW. The significance of contrast blush on computed tomography in children with splenic injuries. *J Pediatr Surg*. 2004 | Retrospective study | 133 | Paediatric population, blush does not mandate SAE | Excluded |
| **37** | Marmery H, Shanmuganathan K, Alexander MT, Mirvis SE. Optimization of selection for nonoperative management of blunt splenic injury: Comparison of MDCT grading systems. *Am J Roentgenol*. 2007 | Retrospective observational study | 496 | Comparison of grading systems | Included |
| 38 | Marmorale C, Guercioni G, Siquini W, et al. Non-operative management of blunt abdominal injuries . *Chir Ital*. 2007 | Retrospective study | 123 | Nonspecific patient group, low statistical power | Excluded |
| 39 | Matsushima K, Kulaylat AN, Won EJ, Stokes AL, Schaefer EW, Frankel HL. Variation in the management of adolescent patients with blunt abdominal solid organ injury between adult versus pediatric trauma centers: an analysis of a statewide trauma database. *J Surg Res*. 2013 | Retrospective study | 1532 | Paediatric study group | Excluded |
| 40 | Mayglothling JA, Haan JM, Scalea TM, J.A. M, J.M. H, T.M. S. Blunt splenic injuries in the adolescent trauma population: The role of angiography and embolization. *J Emerg Med*. 2009 | Retrospective study | 97 | Adolescent study group | Excluded |
| 41 | Mikocka-Walus A, Beevor HC, Gabbe B, Gruen RL, Winnett J, Cameron P. Management of spleen injuries: the current profile. *ANZ J Surg*. 2010 | Retrospective study | 318 | Unrepresentative patient population | Excluded |
| 42 | Miller PR, Chang MC, Hoth JJ, et al. Prospective Trial of Angiography and Embolization for All Grade III to V Blunt Splenic Injuries: Nonoperative Management Success Rate Is Significantly Improved. *J Am Coll Surg*. 2014 | Prospective study | 168 | Prospective use of angiography and SAE | Included |
| **43** | Olthof DCC, Sierink JCC, van Delden OMM, Luitse JSKSK, Goslings JCC. Time to intervention in patients with splenic injury in a Dutch level 1 trauma centre. *Inj J CARE Inj*. 2014 | Retrospective study | 96 | Time to intervention | Included |
| **44** | Olthof DC, Joosse P, Bossuyt PMM, et al. Observation Versus Embolization in Patients with Blunt Splenic Injury After Trauma: A Propensity Score Analysis. *World J Surg*. December 2015 | Propensity score analysis | 206 | Use of propensity score to contemperous patient groups | Included |
| **45** | Olthof DC, van der Vlies CHCH, van der Vlies CHCH, et al. Consensus strategies for the nonoperative management of patients with blunt splenic injury: A Delphi study. *J Trauma Acute Care Surg*. 2013 | Delphi study between 30 experts | N/A |  | Included |
| 46 | P. R, T. G, B. S, et al. Management of blunt injuries to the spleen. *Br J Surg*. 2010 | Retrospective study | 206 | Succes of NOM, age | Included |
| 47 | Parihar ML, Kumar A, Gamanagatti S, et al. Role of splenic artery embolization in management of traumatic splenic injuries: a prospective study. *Indian J Surg*. 2013 | Prospective study | 67 | Prospective study of success rates with NOM | Included |
| 48 | Ransom KJ, Kavic MS. Laparoscopic splenectomy for blunt trauma: a safe operation following embolization. *Surg Endosc OTHER Interv Tech*. 2009 | Retrospective study | 46 | Laparoscopic splenectomy is safe, not considered for this review | Excluded |
| 49 | Requarth JA. Distal Splenic Artery Hemodynamic Changes During Transient Proximal Splenic Artery Occlusion in Blunt Splenic Injury Patients: A Mechanism of Delayed Splenic Hemorrhage. *J Trauma Inj Infect Crit Care*. 2010 | Retrospective study | 7 | Distal versus proximal embolization, lack of statistical power | Excluded |
| 50 | S.-C. W, R.-J. C, A.D. Y, et al. Complications associated with embolization in the treatment of blunt splenic injury. *World J Surg*. 2008 | Retrospective study | 152 | Complications of SAE | Included |
| 51 | Sabe AA, Claridge JA, Rosenblum DI, Lie K, Malangoni MA. The effects of splenic artery embolization on nonoperative management of blunt splenic injury: a 16-year experience. *J Trauma*. 2009 | Retrospective study | 815 | Three groups, more success NOM when combined with SAE | Included |
| 52 | Shih H-C, Wang C-Y, Wen Y-S, et al. Spleen artery embolization aggravates endotoxin hyporesponse of peripheral blood mononuclear cells in patients with spleen injury. *J Trauma*. 2010 | Observational study | 16 | Effect of SAE on splenic function | Included |
| 53 | Shiping L, Jianyong L, Zhi Z, Yun Z. Management of Traumatic Splenic Rupture in Adults: A Single Center’s Experience in Mainland China. *Hepatogastroenterology*. 2014 | Retrospective study | 125 | No full text available | Excluded |
| 54 | Skattum J, Loekke RJV, Titze TL, et al. Preserved function after angioembolisation of splenic injury in children and adolescents: A case control study. *Inj J CARE Inj*. 2014 | Case control | 11 | Pediatric and adolescent study group, case control | Excluded |
| 55 | Soo K-M, Lin T-Y, Chen C-W, et al. More Becomes Less: Management Strategy Has Definitely Changed over the Past Decade of Splenic Injury-A Nationwide Population-Based Study. *Biomed Res Int*. 2015 | Retrospective study | 578 | Lack of statistical analysis, no added value to article | Excluded |
| 56 | Stassen NA, Bhullar I, Cheng JD, et al. Selective nonoperative management of blunt splenic injury: An Eastern Association for the Surgery of Trauma practice management guideline. *J Trauma Acute Care Surg*. 2012 | Guideline | N/A | Guideline | Excluded |
| 57 | Wahl WL, Ahrns KS, Chen S, et al. Blunt splenic injury: Operation versus angiographic embolization. *Surgery*. 2004 | Retrospective study | 164 | Factors to consider for indication of SAE versus operative management | Included |
| 58 | Wei B, Hemmila MR, Arbabi S, et al. Angioembolization reduces operative intervention for blunt splenic injury. *J Trauma - Inj Infect Crit Care*. 2008 | Retrospective study | 317 | less complications and better outcomes with SAE | Included |
| 59 | Wu S-C, Fu C-Y, Muo C-H, Chang Y-J. Splenectomy in trauma patients is associated with an increased risk of postoperative type II diabetes: a nationwide population-based study. *Am J Surg*. 2014 | Retrospective study | 3723 | Increased risk for T2DM | Included |
| 60 | Zarzaur BL, Croce MA, Fabian TC. Variation in the Use of Urgent Splenectomy After Blunt Splenic Injury in Adults. *J TRAUMA-INJURY Infect Crit CARE*. 2011 | Retrospective study | 11.793 | Mortality after splenectomy | Included |
| 61 | Zarzaur BL, Savage SA, Croce MA, Fabian TC. Trauma center angiography use in high-grade blunt splenic injuries: Timing is everything. *J Trauma Acute Care Surg*. 2014 | Retrospective study | 10.405 | Use of angio and role in splenectomy | Included |
| 62 | Zarzaur BL, Kozar R, Myers JG, et al. The splenic injury outcomes trial. *J Trauma Acute Care Surg*. 2015 | Prospective observational study | 383 | Risk of splenectomy after NOM + SAE, importance of blush on CT | Included |
