# Supplementary material for: Splenic artery embolization: technically feasible but not necessarily advantageous
Source: World J Emerg Surg. 2016 Sep 13;11(1):47. doi: 10.1186/s13017-016-0100-7 (PMC5020467; doi:10.1186/s13017-016-0100-7)
Supplement: Additional file 1: Table 1. — Search results and number of articles retrieved after applying the selection criteria.After the initial search, all articles were entered in a reference database (Mendeley). Table 2. Summary table of articles that met inclusion criteria after initial selection. Articles marked in grey were excluded. Table 3. Traditionally used American Association for the Surgery of Trauma (AAST) scoring system for splenic injuries. Figure 1. Algorithm for management of splenic trauma modified from Ekeh and Tugnoli.8, 32 Abbreviations: HD: hemodynamically; BP: blood pressure; FAST: Focused Assessment with Sonography for Trauma; ICU: Intensive Care Unit; SAE: splenic artery embolization; MDCT: Multidetector CT grading (Table 4); NOM: non operative management; CE: contrast extravasation; IV: intravenous. (ZIP 126 kb) [file 13017_2016_100_MOESM1_ESM.zip › Table 1.docx]

**Table 1.** Search results and number of articles retrieved after applying the selection criteria.

After the initial search, all articles were entered in a reference database (Mendeley).

| **Database searched** | **Search terms used and limits applied** | **Number of results** | **Number met inclusion criteria** |
| --- | --- | --- | --- |
| Medline | (("splenic injury") AND "embolization") AND "operative" | 50 | 29 |
| Medline | (((splenic trauma) AND operative management) AND artery embolization) AND non operative management | 15 | 5 |
| Trip database | (title:splenic injury)(title:embolization)(operative) | 8 | 7 |
| EMBASE: Search 1 | 'splenic injury'/exp OR 'splenic injury' AND ('embolization'/exp OR embolization) AND operative | 90 | 46 |
| EMBASE: Search 2 | #1 AND ([adolescent]/lim OR [adult]/lim OR [young adult]/lim) | 46 | 26 |
| EMBASE: Search 3 | #2 AND ('clinical article'/de OR 'clinical trial'/de OR 'comparative study'/de OR 'controlled study'/de OR 'major clinical study'/de OR 'medical record review'/de OR 'multicenter study'/de OR 'observational study'/de OR 'outcomes research'/de OR 'prospective study'/de OR 'retrospective study'/de) AND ('article'/it OR 'review'/it) | 26 | 21 |
| Web of Science | TOPIC: (splenic injury) AND TOPIC: (embolization)AND TOPIC: (operative)  Timespan: All years. Indexes: SCI-EXPANDED, SSCI, A&HCI, CPCI-S, CPCI-SSH. | 91 | 45 |
| Cochrane Library | splenic injury embolization | 7 | 6 |
| Cochrane Library | splenic injury operative | 1 | 0 |
| Scopus | TITLE-ABS-KEY ( splenic injury ) AND TITLE-ABS-KEY ( embolization ) AND TITLE-ABS-KEY ( operative ) | 79 | 34 |
| Manual search from reference list of retrieved articles | Number in reference list   - 1 - 7 - 8 - 9 - 14 - 15 - 16 - 18 - 24 - 30 - 38 - 41 - 42 - 51   Not selected   - Hartnett KL, Winchell RJ, Clark DE (2003) Management of adult splenic injury: a 20-year perspective. Am Surg 69:608–611. - Upadhyaya P (2003) Conservative management of splenic trauma: history and current trends. Pediatr Surg Int 19:617–627. - Todd SR, Arthur M, Newgard C et al (2004) Hospital factors   associated with splenectomy for splenic injury: a national perspective. J Trauma 57:1065–1071.   - Galvan DA, Peitzman AB (2006) Failure of nonoperative management of abdominal solid organ injuries. Curr Opin Crit Care 12:590–594. - Watson GA, Rosengart MR, Zenati MS et al (2006) Nonoperative management of severe BSI: are we getting better? J Trauma 61:1113–1119. - Sclafani SJ, Shaftan GW, Scalea TM et al (1995) Nonoperative salvage of computed tomography-diagnosed splenic injuries: utilization of angiography for triage and embolization for hemostasis. J Trauma 39:818–827. | 22 | 14 |
| TOTAL |  | 435 | 233 |
